# Sialylated Glycan Bindings from SARS-CoV-2 Spike Protein to Blood and Endothelial Cells Govern the Severe Morbidities of COVID-19

**DOI:** 10.3390/ijms242317039

**Published:** 2023-12-01

**Authors:** David E. Scheim, Paola Vottero, Alessandro D. Santin, Allen G. Hirsh

**Affiliations:** 1 US Public Health Service, Commissioned Corps, Inactive Reserve, Blacksburg, VA 24060, USA; 2 Department of Biomedical Engineering, University of Alberta, Edmonton, AB T6G 1Z2, Canada; vottero@ualberta.ca; 3 Department of Obstetrics, Gynecology & Reproductive Sciences, Yale School of Medicine, P.O. Box 208063, New Haven, CT 06520, USA; alessandro.santin@yale.edu; 4 CryoBioPhysica Inc., Chevy Chase, MD 20815, USA; allenhir@earthlink.net

**Keywords:** SARS-CoV-2, spike protein, COVID-19, sialic acid, glycophorin A, hemagglutination, hemagglutinin esterase

## Abstract

Consistent with well-established biochemical properties of coronaviruses, sialylated glycan attachments between SARS-CoV-2 spike protein (SP) and host cells are key to the virus’s pathology. SARS-CoV-2 SP attaches to and aggregates red blood cells (RBCs), as shown in many pre-clinical and clinical studies, causing pulmonary and extrapulmonary microthrombi and hypoxia in severe COVID-19 patients. SARS-CoV-2 SP attachments to the heavily sialylated surfaces of platelets (which, like RBCs, have no ACE2) and endothelial cells (having minimal ACE2) compound this vascular damage. Notably, experimentally induced RBC aggregation in vivo causes the same key morbidities as for severe COVID-19, including microvascular occlusion, blood clots, hypoxia and myocarditis. Key risk factors for COVID-19 morbidity, including older age, diabetes and obesity, are all characterized by markedly increased propensity to RBC clumping. For mammalian species, the degree of clinical susceptibility to COVID-19 correlates to RBC aggregability with *p* = 0.033. Notably, of the five human betacoronaviruses, the two common cold strains express an enzyme that releases glycan attachments, while the deadly SARS, SARS-CoV-2 and MERS do not, although viral loads for COVID-19 and the two common cold infections are similar. These biochemical insights also explain the previously puzzling clinical efficacy of certain generics against COVID-19 and may support the development of future therapeutic strategies for COVID-19 and long COVID patients.

## 1. Introduction

The virus that caused COVID-19 was first named “severe acute respiratory syndrome coronavirus 2” (SARS-CoV-2) in February 2020 in recognition of the disease’s pulmonary symptoms and the lung’s role as its initial target organ, as with its SARS predecessor. Yet as clinical experience and histological findings accrued, the hypoxia which emerged as a key morbidity of severe COVID-19 was found in a large percentage of such patients to accompany nearly normal breathing mechanics and lung gas volume [1,2,3,4,5,6]. Although COVID-19 typically gains infectious penetration in the respiratory epithelium, microvascular occlusion is frequently observed in pulmonary septal capillaries and in other organ systems of COVID-19 patients [7,8,9,10,11,12,13,14,15,16,17,18,19,20], accompanying morbidities such as intravascular clotting and peripheral ischemia [2,3,8,18,21,22,23]. Lung inflammation and other pulmonary symptoms are common with COVID-19, yet in several cases of severe disease, histological examinations have revealed microthrombi and extensively damaged endothelium in the septal capillary microvasculature adjoining relatively intact alveoli [14,24].

Soon after the determination of SARS-CoV-2 as the viral cause of COVID-19, ACE2 was identified as the host cell receptor supporting its replication [25,26,27], with neurophilin-1 its replication receptor for astrocytes and possibly certain other cell types [28,29]. Yet ACE2 is one of a variety of host cell receptors that different coronavirus strains use for replication; other receptors include DPP4 for MERS, APN for HCoV-229E, and CEACAM1 for MHV [30]. The morbidities of SARS-CoV-2, in particular, as shown below, are less dependent on its host cell replication receptor, ACE2, than on glycans having sialic acid (SA) terminal moieties found on viral spike protein (SP) and host cells. For coronaviruses, these sialylated glycans on their SP serve as the initial points of viral attachment to the host cell surface [30,31,32,33,34,35,36,37,38,39,40,41,42], after which the virus can migrate to fuse with a replication receptor [40,42,43,44,45,46,47,48,49]. One clue to the centrality of glycan bindings to the morbidities of the five human betacoronaviruses is the expression by the two common cold strains, HKU1 and OC43, of hemagglutinin esterase (HE), which releases glycan bindings between viral SP and host cells [50,51,52,53,54]. These common cold infections are generally benign, while the SARS, SARS-CoV-2 and MERS viruses do not express HE [50,51,52,53,54] and are deadly, even though the viral loads for COVID-19 and these common cold infections are about the same [55].

### The Molecular Composition of Glycans on SARS-CoV-2 SP and the RBC

The arrangement and chemical composition of the SARS-CoV-2 SP glycans have been determined, with those at its 22 N-glycosylation sites having a total of nine SA terminal residues [31,48,49,56,57,58,59,60,61,62,63] and its four O-glycans having a total of three SA terminal residues [63]. This provides a basis for exploring these viral SP attachments to host cells, notably red blood cells (RBCs), platelets, leukocytes and endothelial cells [31]. RBCs and platelets have densely distributed sialoglycoproteins but no ACE2 receptors on their surfaces [64,65]; the same holds for leukocytes and most other blood cells [66,67,68]. Endothelial cells likewise have a heavily sialylated surface coating (glycocalyx), with about 28,000 SA-tipped CD147 receptors but only about 175 ACE2 receptors per cell [69,70].

**Figure 1:**
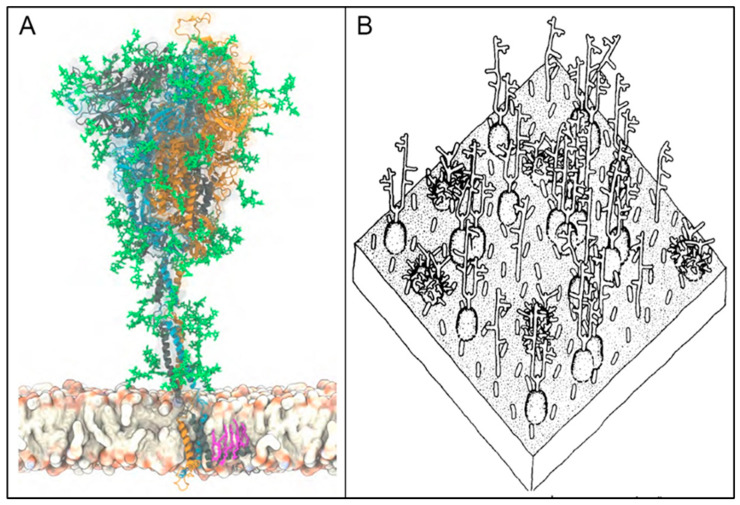
(**A**): Atomistic model of the full-length trimeric S protein of SARS-CoV-2 shown in cartoon representation, reproduced from Sikora et al. (2021) [90]. The three monomeric chains are differentiated by color, with glycans shown in green licorice representation, and a palmitoylated cysteine residue shown in pink, anchored into the viral envelope at the bottom. (**B**): A representation of a 35 × 35 nm area of the RBC surface depicting its sialoglycoprotein coating, reproduced from Viitala et al. (1975) [71]. Prominent among these sialylated glycans are GPA strands, which extend approximately 5 nm from the RBC surface, and band 3 protein, which extends > 10 nm from that surface and is glycosylated by poly-N-acetyllactosamine. Reproduced (**A**) under CC-BY 4.0 and (**B**) with permission from Elsevier.

Of particular interest are attachments of SARS-CoV-2 SP to the RBC, the latter coated with one million SA-tipped glycophorin A (GPA) molecules and a total of 35 million SA monosaccharides per cell [71,72,73]. The heavily sialylated GPA strands are spaced about 14 nm apart on the RBC surface and extend out 5 nm [71]. Band 3 protein is another molecule on the RBC surface, with 1.2 million copies per RBC, which extends > 10 nm from the RBC surface [71,74] and is glycosylated by poly-N-acetyllactosamine, a sialylated branched-chain glycan [75,76,77,78]. GPA and poly-N-acetyllactosamine, the two most abundant glycans on the RBC membrane [77], have been found to mediate hemagglutination by various bacterial and viral pathogens [78,79,80,81]. The glycans attached to SARS-CoV-2 SP and those which extend from the RBC surface are depicted in Figure 1.

Hemagglutination as caused by these pathogen–glycan attachments is of particular interest in view of a primal defense mounted by RBCs along with platelets against pathogens having SA terminal moieties by attaching to them and delivering them to leukocytes or conveying them to macrophages in the liver and spleen for phagocytosis [72,82,83,84,85,86,87,88]. Notably, GPA, one of the two most abundant glycans on the RBC surface [77,89], has no other known physiological role other than spearheading this pathogen defense [71,72,83,84]. For severe COVID-19 infections, however, this primal defense, described as “immune adherence” [85], goes self-destructively overboard, with the total load and sizes of clumps formed exceeding the body’s capacity to sequester them, as detailed below.

A clear experimental demonstration of binding between SARS-CoV-2 SP and sialylated glycans on host cells was provided using NMR spectroscopy [34]. It was found, in particular, that a site on the SP N-terminal domain (NTD) binds to α2,3 and α2,6 sialyl N-acetyllactosamine, which are components or variants thereof of the sialylated poly-N-acetyllactosamine glycans of the band 3 strands extending from the RBC surface. Intriguingly, this SP-to-glycan binding was found to be much more pronounced for α2,3 than for α2,6 SA-linked N-acetyllactosamine [34], while α2,3 vs. α2,6-linked SA is likewise much more prevalent in sialylated poly-N-acetyllactosamine of adult (vs. fetal) RBCs [76].

**Figure 2:**
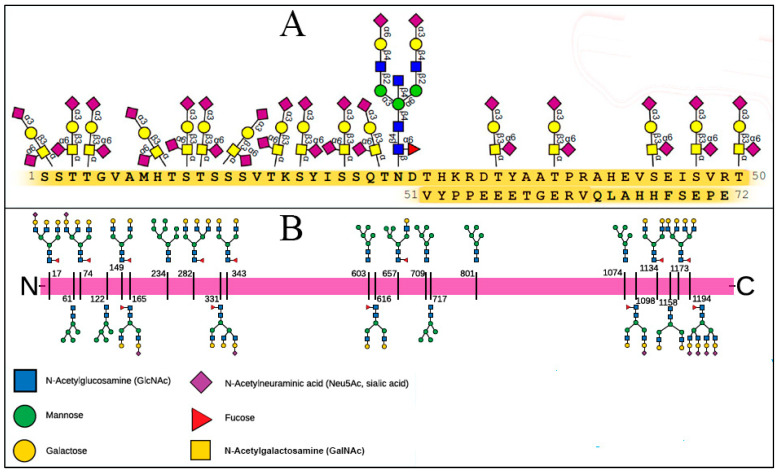
(**A**): Amino acid sequence of the extracellular domain (aa 1–72) of GPA with its glycan structures and attachment sites, adapted from Jaskiewicz et al. (2019) [94]. (**B**): The terminal monosaccharides for fully populated N-glycans of a SARS-CoV-2 SP monomer, with these 22 N-glycosylation sites numbered from the N-terminal end to the C-terminal end, as adapted from Sikora et al. (2021) [90]. The key to the monosaccharides shown in both (**A**) and (**B**) is at bottom of (**B**). Reproduced (**A**,**B**) under CC-BY 4.0.

Possibilities for binding are indicated as well between SARS-CoV-2 SP and/or glycans at its glycosylation sites and GPA on the RBC surface, with GPA, as noted, having no known physiological role other than this type of immune adherence. The positive electrostatic potential of SARS-CoV-2 SP [91] supports its binding to the negatively charged, densely distributed SA on the RBC surface, most on its million GPA strands [92,93]. Also, as depicted in Figure 2, SA in its predominant human form, Neu5Ac, is the most common terminal residue of GPA [71,74,94]. For the N- and O-glycans on SARS-CoV-2 SP, the most common terminal residues are galactose (Gal), with 27 total, and Neu5Ac (SA), with 12 total [61,62,63,90]. Through binding configurations proposed by Varki and Schnaar (2017) [95] and others [34,96,97,98], multivalent bonds can form via α2–3 and α2–6 linkages from Neu5Ac on GPA to Gal on glycans populating SARS-CoV-2 SP glycosylation sites.

## 2. In Vitro, In Vivo and Clinical Studies Demonstrate Induction of RBC Aggregation by SARS-CoV-2 SP

Many in vitro, in vivo and clinical studies demonstrate that SARS-CoV-2 SP attaches to RBCs and induces RBC aggregation. Boschi et al. (2022) found that SARS-CoV-2 SP from each of the Wuhan, Alpha, Delta and Omicron strains induced RBC clumping (hemagglutination) when mixed with human RBCs in phosphate-buffered saline (PBS) [91]. To explore whether bridging of adjacent RBCs by SARS-CoV-2 SP via glycan bonds might be the cause of this observed hemagglutination, an agent with indicated high-affinity binding to multiple SARS-CoV-2 SP glycan-binding sites [99], the macrocyclic lactone ivermectin (IVM), was added to the mix of SP and RBCs both before and after hemagglutination formed. IVM blocked the formation of hemagglutination when added to the initial mix and reversed hemagglutination over the course of 30 min when added after it formed [91]. In another study, SARS-CoV-2 SP added to whole blood induced clumping of RBCs, hyperactivation and clumping of platelets, and formation of anomalous fibrinogen deposits [100].

**Figure 3:**
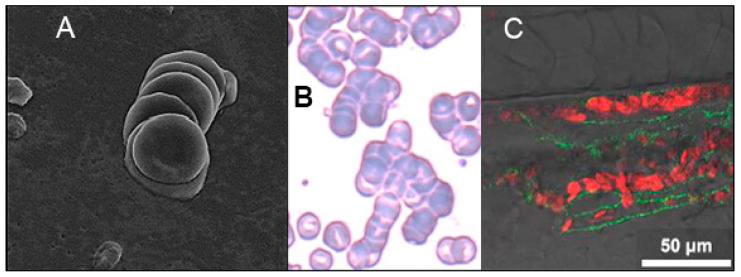
(**A**,**B**): Images of RBC rouleaux (stacked clumps) from the blood of COVID-19 patients, obtained using electron (magnification ×5000) [106] and light (80× objective) [107] microscopy. The first study (**A**) found RBC clumps in all 31 patients studied, all with mild COVID-19 [106], and the second (**B**) found large RBC aggregates in 85% of COVID-19 patients with anemia [107]. (**C**): A frame from a video of RBC aggregates in capillaries of zebrafish embryos that formed within 3–5 min after injection of SARS-CoV-2 SP into the common cardinal vein at a similar concentration to that obtained in critically ill COVID-19 patients [103]. The velocity of blood flow in the capillaries shown in this video frame was markedly reduced from that prior to injection of SP. Reproduced (**A**) with permission from Georg Thieme Verlag KG; (**B**) under CC-BY 4.0; (**C**) with permission from Elsevier.

The same SP-induced RBC clumping effect as noted above was demonstrated in zebrafish embryos, which have blood cell glycosylation patterns [101] and capillary diameters [102] similar to those of humans. When SARS-CoV-2 SP was microinjected into the common cardinal vein of a zebrafish embryo at a concentration similar to that obtained in critically ill COVID-19 patients, it caused the formation of small RBC clumps and an associated reduction in blood flow velocity within 3–5 min after injection, as shown in Figure 3C, accompanied by thrombosis in capillaries, arteries and veins [103]. When SP was coinjected with a mixture of heparan sulfate and heparin (molecular mass of each ≤ 30 kDa), however, with both of these glycosaminoglycans having strong binding affinity to SARS-CoV-2 SP [103,104,105], the extent of thrombosis was markedly reduced [103].

In various studies, SARS-CoV-2 SP and subunits were observed in the plasma of 64% of COVID-19 patients [108], in the sera of 30% of hospitalized COVID-19 patients [109] and in the brains of all of the 13 patients who died of this disease [110]. SP and spike S1 subunits were likewise observed over periods of months in the plasma [111,112,113] and monocytes [114], respectively, of patients having post-acute sequelae of this disease (long COVID, or PASC). Leakage of SP outside of an infected host cell during SARS-CoV-2 replication has been documented in vitro and clinically [115,116,117] and may be the source of the SP in blood found in COVID-19 patients.

SARS-CoV-2 SP attachments to RBCs were demonstrated directly by Lam et al. (2021) through immunofluorescence analysis of RBCs from the blood of nine hospitalized COVID-19 patients [118]. For these patients at hospital admission, the mean percentage of RBCs having SARS-CoV-2 SP traces was 41%. This finding suggests that concentrations of SARS-CoV-2 SP in blood as typically reported in other studies using plasma or serum may significantly understate actual values due to high-affinity binding to RBCs, which are removed from plasma and serum. SARS-CoV-2 SP and pseudovirus were each found to bind to nanoparticle arrays bearing SA derivatives [59] and to SA-tipped CD147 receptors, likewise detected using nanoarrays [119]. Nanoarray methods are required to detect SARS-CoV-2 SP glycan attachments, because these methods allow bindings to form multivalently, whereas univalent bindings are weak [31] and not detectable by microarray methods [42]. Studies using the latter failed to detect SARS-CoV-2 SP bindings to either SA [120] or CD147 [121]. As noted above, binding of SARS-CoV-2 SP to sialylated glycans identical or closely related to those on the RBC surface was demonstrated directly using NMR spectroscopy [34].

The presence of SARS-CoV-2 SP in the blood of COVID-19 patients and its induction of hemagglutination in vitro and in vivo would suggest that the same would occur clinically, which is indeed the case. In three publications that used scanning electron microscopy to examine blood from the cubital vein blood of patients with mild-to-moderate cases of COVID-19, all hospitalized but none requiring intensive care, a team of investigators observed blood cell clumping and other anomalies [106,122,123]. The first study found stacked RBC aggregates (rouleaux) ranging in size from 3–12 cells, as shown in Figure 3A, in the blood of all 31 of its COVID-19 patients, with none found in 32 matched healthy controls [106]. A follow-up publication reported the mean count of RBC aggregates in the COVID-19 patients at 3.1 to 5.5 per 1000 µm^2^ scanning area, while controls had no RBC aggregates [122]. Aggregates of platelets, some with leukocytes or RBCs, were likewise found in all COVID-19 patients to significant extents, but none were found in the controls [122].

Light microscopy examination of smears from the blood of 20 hospitalized COVID-19 patients with anemia detected large, stacked RBC clumps (rouleaux), as shown in Figure 3B, in 85% of those patients [107]. Another study, which examined the sublingual microcirculation of 38 COVID-19 patients in intensive care using video microscopy, found that the mean number of RBC microaggregates detected in these patients was 15 times the mean number for 33 healthy volunteers [124]. These RBC microaggregates were found in two-thirds of the COVID-19 patients vs. two of the 33 healthy volunteers. A study of the blood of 172 hospitalized COVID-19 patients found that both RBC aggregability and the strength of RBC aggregates formed were significantly greater than those values for healthy controls and that this RBC hyperaggregability correlated with enhanced blood coagulation, all of these effects highly significant (*p* < 0.001) [125]. The much greater degree and strength of RBC aggregation found in COVID-19 vs. sepsis, with both having elevated levels of inflammation-related markers, indicate that inflammation alone cannot explain these RBC aggregation effects for COVID-19 [125].

Paralleling these studies that document RBC aggregation in severe COVID-19 are many that report microvascular occlusion. Postmortem examinations of hundreds of patients who died from COVID-19 in many studies consistently found microthrombi in the pulmonary microvasculature in most patients [7,8,9,10,11,12,13,14,15,16,17,18]. Microthrombi in alveolar capillaries were nine times as prevalent in postmortem COVID-19 patients compared to influenza patients [10]. RBC clumping and microthrombi in the lungs have been regarded as likely causes of hypoxemia in severe COVID-19 patients [1,2,106,123], which in turn is closely associated with mortal outcomes [126].

Microthrombi elsewhere in the body, including in the heart, kidneys and liver, were also frequently observed in autopsy examinations of COVID-19 patients, with indications that these may have contributed to multiorgan damage and failure [7,8,20]. Another indication of the widespread distribution of microthrombi throughout the body in severe COVID-19 patients, persisting even after recovery from acute illness, was provided using video capillaroscopy to examine ocular conjunctival microvessels in 17 hospitalized COVID-19 patients within 28 days after hospital discharge and 17 healthy controls [127]. The mean percentage of occluded microvessels was found to be six times as high in the hospital-discharged COVID-19 patients vs. controls, while the mean rates of blood flow in the conjunctival capillaries and postcapillary venules were significantly lower [127]. Such widespread indications of microvascular occlusion in severe COVID-19 patients led cardiovascular researchers at the Johns Hopkins and Harvard University medical schools to conclude that “severe COVID-19 is a microvascular disease” [21].

## 3. Glycan Bindings from SARS-CoV-2 SP to Platelets and Endothelial Cells Cause Endothelial Damage, Inflammation and Coagulation

Attachments of SARS-CoV-2 SP to the heavily sialylated [64,65,70] surfaces of platelets and endothelial cells cause endothelial damage, platelet activation and associated coagulation which, as with the attachments to RBCs, contribute to the severe morbidities of COVID-19. Platelets, having no ACE2 receptors, like RBCs [66,67], act with RBCs in a role that was termed “immune adherence” [85], attaching to and clearing pathogens [87,88], and are found enmeshed with RBCs in blood cell clumps in COVID-19 patients [122]. The degree of sialyation of the endothelial cell surface is exemplified by the 28,000 SA-tipped CD147 receptors vs. the 175 ACE2 receptors per endothelial cell [69]. For glomerular endothelial cells from a conditionally immortalized human cell line, the enzyme neuraminidase, which hydrolyzes SA, removed more than 50% of the cells’ surface coating (glycocalyx) [70]. The endothelial cell thus provides a prime target for the SARS-CoV-2 virus, and indeed, both whole virus and viral SP have been found on endothelial cells in clinical and in vivo COVID-19 infections [10,17,24,110,128,129,130,131]. Correspondingly, damaged endothelial cells have been frequently observed in severe COVID-19 patients [21,24,132,133]. Yet the importance of this direct viral attack on the endothelium in COVID-19 has been overlooked by some researchers in the belief that ACE2, which is sparse on endothelial cells, is the only host-cell binding target of interest for SARS-CoV-2 [134,135].

These SARS-CoV-2 viral or SP attachments to the endothelium can be perilous to the human host, with trillions of RBCs each flowing once per minute through the lungs and then the extrapulmonary vasculature [136] and with the cross-sectional diameter of most capillaries so small that RBCs distort their shape to squeeze through [137]. Thus, SARS-CoV-2 virus particles or SP attached to endothelial cells or RBCs could create resistance to blood flow or even potentially rip off a piece of an endothelial cell or the entire cell [31]. Indeed, one study found that serum levels of circulating endothelial cells (CECs) in mild-to-moderate COVID-19 patients were up to 100 times the levels for matched controls. The study also found that each of these CECs from the COVID-19 patients typically had several holes in their membranes approximately the size of the SARS-CoV-2 viral capsid (the viral envelope) [106]. A marker of endothelial damage, von Willebrand factor (VWF), which promotes platelet activation and, in turn, coagulation [138,139,140], has been found to be significantly elevated in COVID-19 patients [21,132,141,142]. These and other coagulation and proinflammatory pathways can cause blood clots or trigger a cytokine storm in the most serious cases of this infection [21,132,133].

While these pathological pathways contribute significantly to the severe morbidities of COVID-19, the role of SARS-CoV-2 SP-induced RBC aggregation in these morbidities is nevertheless central, as demonstrated below through multiple avenues of substantiation. We show below that experimentally induced RBC clumping in vivo causes the same morbidities and the same redistribution of blood flow from smaller to larger blood vessels as for COVID-19. We further demonstrate the following: (i) key risk factors for COVID-19 morbidity are associated with markedly increased RBC aggregation; (ii) SARS-CoV-2 SP in the absence of whole virus induces microvascular occlusion in vivo and clinically; (iii) three generic drugs that have aroused widespread interest as potential COVID-19 treatments all significantly inhibit RBC aggregation; and (iv) for mammalian species, the degree of clinical susceptibility to COVID-19 correlates to aggregation propensity of RBCs with *p* = 0.033.

## 4. Experimentally Induced RBC Clumping In Vivo: Parallels to Severe COVID-19

Studies dating back to the 1940s in dogs, rabbits, mice, hamsters and other animals closely examined the effects of IV injection of high-molecular-weight dextran (HMWD), generally of molecular weight (MW, loosely equivalent to molecular mass) ≥ 100 kDa or other blood cell-agglutinating agents. In several studies, blood cell aggregation was induced within minutes to hours after IV injection of HMWD [143,144,145,146,147,148], with molecular bridging of RBCs by HMWD molecules being a hypothesized mechanism for this effect [149,150,151,152]. After HMWD injection in vivo, small clumps of RBCs formed and then enlarged into longer stacked clumps (rouleaux) and, in some cases, into vast trees with branches of hundreds of stacked RBCs [144,145,153]. Also, the addition of low-MW dextran (LMWD, e.g., MW ≤ 40 kDa) in vivo prevented the formation of RBC aggregates when injected with HMWD [146,154] and rapidly disaggregated them with accompanying reversal of microvascular occlusion when injected after HMWD-induced clumps had formed [148,155,156,157,158].

In vitro, the addition of HMWD to blood likewise induced RBC aggregation [159,160] and did so as well when added to RBCs in PBS [161,162]. The same RBC disaggregating effect of LMWD was observed in vitro [163], possibly caused by competitive binding to RBCs that limited bridging between adjacent RBCs by larger molecules. Although we have focused on aggregation of RBCs, these same aggregating effects of HMWD and disaggregating effects of LMWD have been observed, both in vitro and in vivo, for platelets as well [147,153].

Even in healthy humans or animals, RBC clumps can transiently form under conditions of slow blood flow, e.g., in deep veins of the lower limbs, but they typically disaggregate as they move into regions of faster blood flow [164,165,166,167,168,169,170,171,172,173,174] and are rarely problematical in healthy subjects [148,157,175]. Yet under pathological conditions in diseases such as diabetes, malignant hypertension and malaria [154,167,175,176,177], these RBC aggregates can persist and grow via a positive feedback loop whereby the clumps cause decreased blood flow velocity with a concomitant reduction in shear forces that in turn causes further aggregation [164,166,167,168,169,170,171,173,175]. In mammals, a significant total mass of blood cell aggregates can lodge in a distributed network of arterioles before obstruction of blood flow reaches a critical stage [178]. Pulmonary artery tips provide a catch-trap architecture that sequesters large blood cell aggregates, which limits disseminated microvascular occlusion and mitigates resulting hypoxia and associated widespread tissue damage, including to the heart wall [167,178].

The capability of LMWD to rapidly reverse RBC aggregation and associated microvascular occlusion caused by injection of HMWD, as noted above, distinguishes blood clumping, e.g., as induced by HMWD, from clotting, in which blood cell clumps harden into fibrin-enmeshed clots via the coagulation cascade. Indeed, several mammalian diseases are associated with increased levels of RBC aggregation and microvascular occlusion which do not typically cause blood clotting, although risks of this complication are increased [154,175]. Blood cell clumping and clotting are not completely unrelated phenomena, however, given the potential of RBC aggregation to trigger deep vein thrombosis [179,180] and the role of fibrinogen, an essential promoter of blood clotting, in blood cell clumping as well [164,181,182,183].

### 4.1. Induced RBC Aggregation Causes Microvascular Occlusion, Hypoxia, Blood Clots, and Redistribution of Blood Flow from Smaller to Larger Blood Vessels

When HMWD or other agglutinating agents were injected into animals at sufficient concentrations to overwhelm the host’s ability to safely sequester the RBC aggregates formed, these clumps caused microvascular occlusion as detected in a variety of host tissues [154], including the myocardium [153,184], muscle [185] and abdominal cavity [153] of rats; the conjunctival vessels of dogs, cats and rabbits [147,186]; the cheek pouch of hamsters [148,157]; and the kidney, liver, ear chamber, bone marrow and heart tissue of rabbits, including the myocardium and pericardium [144,145,146,155,156]. In the myocardium of rabbits and rats, the degree of myocardial tissue damage was correlated with the observed degree of intravascular aggregation of blood cells [144,146,153], with hypoxia resulting from vascular occlusion proposed to be the cause of tissue damage [144,146].

Associated with the microvascular occlusion that it triggered, experimentally induced RBC aggregation caused decreased velocity of blood flow [143,145,146,147,148,154,171,184], increased blood viscosity [143,154,186,187], increased incidence of blood clotting [144,154,167] and decreased oxygen tension in arteries, veins and tissues, with accompanying hypoxic damage to body organs [144,146,154,188,189]. Another effect caused by induced blood cell clumping as observed in the conjunctiva of cats, dogs and rabbits and bone marrow of rabbits was a reduction in blood flow in the capillaries and other small vessels having cross-sectional diameters of about 10 μm or smaller [147,155], indicative of a shift of blood flow into the larger vessels. A similar redistribution of blood flow from the smaller blood vessels of micrometer cross-sectional diameter to larger blood vessels was observed in patients with type II diabetes [177,190], a disease characterized by an increased extent of RBC aggregation and accompanying microvascular occlusion [167,177,191,192,193,194].

### 4.2. Corresponding Morbidities in Severe COVID-19

As considered above, SARS-CoV-2 SP, like HMWD dextran, induces RBC aggregation, and the same morbidities caused by experimentally induced RBC aggregation have been commonly observed for cases of severe COVID-19. These morbidities of severe COVID-19 include microvascular occlusion in the lungs and other organ systems [7,8,9,10,11,12,13,14,15,16,17,18,19,20], hypoxia [1,195], arterial and venous thromboembolisms [9,15,17,18,21,196,197,198], disseminated intravascular coagulation [15,21,196,197,198,199,200] and multiorgan damage associated with these vascular aberrations and hypoxia [7,200,201]. Decreased oxygen saturation is a particularly dangerous morbidity of COVID-19, with a peripheral oxygen saturation (SpO2) of <88% associated with a 3.7-fold increased risk of death [126] and an SpO2 of ≤93% deemed to be a sufficient condition for classifying a COVID-19 infection as severe according to U.S. National Institutes of Health guidelines [202].

### 4.3. Redistribution of Blood Flow from Smaller to Larger Blood Microvessels in COVID-19 Patients

**Figure 4:**
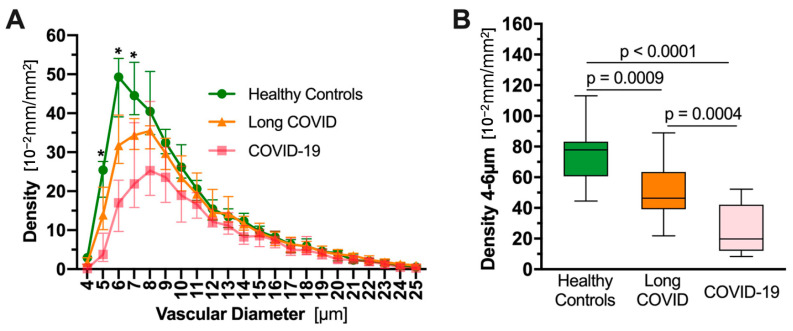
Density of functional capillaries (with flowing RBC content ≥ 50%) of cross-sectional diameter 4–25 μm in the sublingua of long and active, hospitalized COVID-19 patients and healthy controls. (**A**): Functional capillary density by diameter; * denotes *q* < 0.05 (*q* per Storey-Tibshirani). (**B**): Functional capillary density for capillaries of diameter 4–6 μm. Mean values for healthy controls and long and active COVID-19 patients were 77.9, 46.4 and 19.9, respectively, with *p* < 0.001 for comparisons between each pair of patient groups. Reproduced from Osiaevi et al. (2023) [203] (CC-BY 4.0).

Another effect of experimentally induced RBC aggregation, the redistribution of blood flow from microvessels to blood vessels of larger cross-sectional diameter, as described above, is also paralleled in COVID-19 is. Osiaevi et al. (2023) compared videomicroscopic imaging of the sublingual microvasculature of 16 critically ill COVID-19 patients, 17 patients with long COVID and 15 healthy controls [203]. As shown in Figure 4, the density of functional capillaries (having flowing RBC content ≥ 50%) with cross-sectional diameter 4–10 μm was sharply reduced for active COVID-19 patients vs. controls, with values for long COVID patients roughly halfway between those for active COVID-19 patients and healthy controls. The study investigators concluded from these and other measures of microvascular health that the long COVID patients had significant microvasculature impairment, lasting even 18 months after infection for some [203].

Rovas et al. (2021) reported similar sharp reductions in densities of functional capillaries at the lower end of the 4–25 μm cross-sectional diameter range in the sublingual microvasculature of COVID-19 patients vs. healthy controls [201]. The extent of reduction in density of functional capillaries of diameter 4–6 μm in the COVID-19 patients correlated with their oxygenation index (PaO_2_/FiO_2_) and with an index of multiorgan failure and associated mortality risk. Rovas et al. concluded from these correlations that the observed reduction in sublingual small capillary density was another manifestation of the pathological clogging of capillaries as also observed in pulmonary microthrombi at autopsies of COVID-19 patients. A similar marked shift in blood flow from smaller to larger vessels in active [204,205,206,207] and long [208] COVID-19 patients was also observed in blood vessels of larger cross-sectional diameter, 1 mm and greater, using high-resolution CT scans.

Further insights into the prevalence of microvascular occlusion in both active and long COVID-19 were provided by studies that imaged the ocular conjunctiva and retina in human subjects using noninvasive techniques. As noted previously, the percentage of occluded microvessels in the conjunctiva was found to be six times as high in hospital-discharged COVID-19 patients vs. healthy controls [209], while other studies reported that RBC aggregation in the conjunctiva correlated closely with measures of that elsewhere in the body [158,175]. Three studies of perfusion density in various retinal capillary layers found small (e.g., 3–4%) but statistically significant differences (e.g., *p* = 0.011, *p* = 0.04, *p* = 0.003) for COVID-19 patients one month after recovery [210,211] and for long COVID patients [212] vs. healthy controls. Retinal capillary perfusion density was determined with optical coherence tomography angiography (OCT-A), which uses noninvasive laser imaging of RBC flow in retinal capillaries to detect perfusion aberrations.

## 5. Major Risk Factors of Age, Diabetes and Obesity for COVID-19 Severity Correlate with Increased Propensity to RBC Aggregation

The most significant risk factor for severe COVID-19 is age, with several studies showing a multifold increased risk of fatal outcomes with older age [195,213,214]. One multivariate analysis of 17 million subjects in the UK reported a sixfold increased mortality for ages 70 through 79 vs. 50 through 59 years [215]. A meta-analysis of 612,000 subjects in several countries conducted in 2020 found a mortality rate of 22.8% for ages 70–79 years vs. 0.3% for ages ≤ 29 years [216]. Note that the risk factor data considered in this section are for pre-Omicron variants of SARS-CoV-2. Since Omicron variants do not penetrate deeply into the lungs or bloodstream and cause less severe illness than prior variants, as considered in the Discussion section, risk factors for Omicron infections are not necessarily the same as those for pre-Omicron variants nor is the efficacy of various therapeutics.

This multifold increase in COVID-19 mortality with older age aligns with a much greater extent of microvascular occlusion in older vs. younger healthy subjects, linked to both a significantly greater propensity to RBC aggregation and slower blood flow with increased age. Microscopic examinations of the bulbar conjunctiva of healthy subjects found that 30% of those of ages 56–75 years had aggregation in the smaller venules and capillaries, as compared with a 3% rate of such aggregation of those of ages 16–35 years [190]. This tenfold increased rate of microvascular occlusion in the older subjects corresponds to much greater RBC aggregation and slower blood flow with increased age. One study that measured RBC aggregability by multiple detection methods found a statistically significant increase in this value in the blood of middle-aged versus young adults [217]. Another study found highly significant (*p* < 0.001) increases in RBC aggregability and average RBC aggregate size for subjects of ages 66–89 vs. those of 20–30 years [218]. Both of these studies measured RBC aggregability in vitro using drawn blood.

As noted, RBC aggregate formation in vivo depends not only on aggregability under static conditions but also on the degree of shear forces that promote disaggregation, as associated with velocity of blood flow [164,168,169,170]. It is, thus, noteworthy that blood flow is slower with increased age [219,220,221,222,223,224,225]. Mean velocity of capillary flow under fingernail and toenails for subjects of mean age 63 years was half of that for subjects of mean age 26 years [219]. Older subjects had 23% [220] and 40% [221] diminished flow velocities vs. younger subjects for capillary flow in other tissues. Arterial blood flow velocities were 26–27% lower for older vs. younger subjects in two studies [223,224]. The combined effects of increased RBC aggregability and decreased blood flow velocity would appear to account for the tenfold incidence of microvascular occlusion in smaller venules and capillaries of the bulbar conjunctiva with increased age, as noted above.

In a multivariate analysis of COVID-19 risk factors for 17 million patients in the UK, mortality was increased with hazard ratios of 1.31 for diabetics with good glucose control, 1.95 for diabetics with poor glucose control and 1.92 for obesity [215]. An umbrella review of 32 high- or moderate-quality reviews reported odds ratios for mortality of 2.09 for diabetes and 2.18 for obesity [226]. A significant degree of RBC aggregation is characteristic of diabetes [167,177,191,193,194], with this effect especially pronounced for type 1 disease [191] and for diabetics with poor glycemic control [193]. In studies of RBC attributes for subjects of varying body mass index (BMI), BMI correlated with RBC adhesiveness/aggregability at *p* < 0.001, while obese subjects had larger RBC aggregates (*p* < 0.009) that were more resistant to dispersion by flow [227,228]. In summary, three major risk factors for severe COVID-19—increased age, diabetes and obesity—were all characterized by increased RBC aggregability, with this correlation especially striking for age.

## 6. SARS-CoV-2 SP Unattached to Whole Virus Induces Microvascular Occlusion In Vivo

Akin to the studies noted previously demonstrating induction of RBC clumping by SARS-CoV-2 SP in vitro [91,100,229] and in vivo [103], other studies likewise demonstrate that SARS-CoV-2 SP in the absence of whole virus caused microvascular occlusion.

### 6.1. Myocardial Damage as a Signal of Microvascular Occlusion

A clinical window into morbidities associated with RBC aggregation is provided by the myocardium—the heart muscle—which is among the tissues most susceptible to the damaging effects of experimentally induced RBC aggregation and ensuing microvascular occlusion. Several studies found that injection of HMWD (high-MW dextran) caused myocardial damage [144,146,154,230] and/or electrocardiogram (ECG) changes [153,154,187,230] characteristic of myocarditis. In one study, 40 min after HMWD injection, ECG abnormalities were apparent, and HMWD induced lasting myocardial damage [230]. Both the degree of myocardial damage [144,146] and of ECG abnormalities [153] correlated with the extent of microvascular occlusion. Clinically, for hospitalized patients with coronary heart disease, the number of microthrombi per field of observation in the bulbar conjunctival microcirculation was found to be correlated with both the extent of ECG and symptomatic abnormalities [153].

### 6.2. Myocardial Damage Experimentally Induced by SARS-CoV-2 SP in the Absence of Whole Virus

Induction of myocarditis by SARS-CoV-2 SP in the absence of whole virus was evidenced in two rodent studies by IV injection of BNT162b2, the Pfizer-BioNTech mRNA vaccine, an experimental system in which SP is generated by host cells, distinct from intramuscular (IM) injection used for clinically administered COVID-19 vaccinations. Clinical cases of SARS-CoV-2 SP found in endothelial cells after IV mRNA vaccination [231,232,233] support the possibility that SP could be generated by nucleated endothelial cells in blood vessels post-vaccination. In mice, after a second IV vaccine dose, 67% had grossly visible white patches over the visceral pericardium and all showed changes of myopericarditis, compared with only mild degenerative changes in the myocardium in the intramuscular (IM)-injection group [234]. All of the mice in the IV-injection and the IM-injection groups had myocardial WBC infiltration and cardiomyocyte degeneration and necrosis vs. none in saline-injection controls. Rats given two IV doses of BNT162b2 vaccine two weeks apart in another study manifested marked blood hypercoagulability along with apoptotic cardiac muscle fibers, ECG changes and other abnormalities that reflected myocardial injury [235].

### 6.3. Clinical Signs of Microvascular Occlusion and Myocarditis after Exposure to SARS-CoV-2 SP

Further insights into microvascular occlusion caused by SARS-CoV-2 SP in the absence of whole virus in a clinical setting were provided by optical coherence tomography angiography (OCT-A) imaging of the retinal microvasculature. Determinations of the vascular density (VD) of flowing blood vessels in various retinal layers of human subjects, an indicator of microvascular occlusion, found that the CoronaVac vaccine, made from inactivated whole virus, caused no changes after vaccination [236,237]. The Pfizer-BioNTech mRNA vaccine caused small but statistically significant reductions in VD vs. controls at three days [238] and at two and four weeks [237] after vaccination. Reductions in many of these VD values at two weeks after vaccination were statistically significant at *p* < 0.001; most of these resolved by four weeks after vaccination, but seven of these VD reductions persisted at statistically significant levels at that time [237].

The significance of these findings derives not from the occasional ocular adverse effects that have been reported after mRNA COVID-19 vaccinations [239,240] but rather from indications that ocular microvascular occlusion mirrors a pathology elsewhere in the body [158,175]. Myocardial injury is another indicator of microvascular occlusion, as noted above, which opens another diagnostic window, PET-CT scanning, since fluorodeoxyglucose F18 (FDG) uptake in myocardial tissue has been found to track myocardial injury [241,242]. In one study, 700 SARS-CoV-2-vaccinated and 303 nonvaccinated subjects were given PET/CT scans either to evaluate malignancies or perform other medical screenings unrelated to COVID-19 or myocarditis. In PET/CT scans taken 1−180 days after vaccination, myocardial FDG uptake was significantly higher as compared to that for unvaccinated subjects (median of 4.8 vs. 3.3, *p* < 0.0001) [243]. Similar potential risks at a less than clinically overt level were indicated from cardiac test markers 2–10 weeks after COVID-19 mRNA vaccinations vs. pre-vaccination values in 566 patients at a cardiac clinic, with an increase in the 5-year predicted risk of acute cardiac events from 11% to 25% [244].

Whether the clinical indicator is the rare incidence of myocarditis following COVID mRNA vaccinations [245,246] or the greater incidence of cardiac irregularities following such vaccinations, e.g., 1–7% rates of chest pains and abnormal ECG readings in two post-COVID vaccination studies in adolescents [247,248], an association with the presence of SARS-CoV-2 SP in such adverse events is indicated. A study conducted in the US in Boston-area hospitals found that of 16 patients hospitalized for myocarditis after COVID-19 mRNA vaccinations, all had significant levels of SARS-CoV-2 SP unbound by antibodies in blood, whereas 45 asymptomatic, vaccinated subjects had no detectable SP [249]. Investigators at the same hospitals found indications that SARS-CoV-2 mRNA vaccines routinely persist up to 30 days following vaccination and are detectable in the heart [250]. SARS-CoV-2 SP was found on cardiomyocytes of 9 of 15 mRNA-vaccinated subjects with symptoms of myocarditis in another clinical series [251].

## 7. Decreased Clinical Severity of COVID-19 by Agents That Inhibit RBC Aggregation

Analogous to the activity of LWMD (MW ≤ 40 kDa) in limiting and reversing induced RBC aggregation, as noted above, various forms of heparin and heparan sulfate, glycosaminoglycans of MW ≤ 30 kDa, have shown benefits by clinical or laboratory criteria for COVID-19 in a scattering of clinical studies. The specific agents used were subcutaneous heparin plus enoxaparin (low-MW heparin) [252], enoxaparin [122] and a low-MW mixture of 80% heparan sulfate and 20% dermatan sulfate (sulodexide) [123,253]. As noted previously, both heparin and heparan sulfate bind strongly to SARS-CoV-2 SP [103,104,105].

Of particular interest as potential treatments for COVID-19 are three generic drugs which have been closely studied and have received wide attention.

### 7.1. Fluvoxamine

Fluvoxamine (FLV), a selective serotonin reuptake inhibitor (SSRI), attracted interest from prominent medical researchers [254,255,256] after early clinical trials indicated promising results for COVID-19 treatment [257,258,259,260]. Although rapid recovery from severe illness was not generally observed, one study showed a significant reduction in residual symptoms of COVID-19 at 14 days after start of FLV treatment vs. untreated controls [257], and another showed significant reductions in emergency room visits or hospitalizations [259]. Yet the puzzling question raised by these indications of clinical activity was by what biochemical mechanism could an SSRI used to treat depression and anxiety disorders offer therapeutic benefits against a viral disease?

A plausible biochemical mechanism is the sharp reduction by FLV in serum levels of serotonin, which is a powerful inducer of RBC and platelet aggregation. In vitro, serotonin caused marked aggregation of RBCs, platelets and leukocytes [147]. In vivo, injection of serotonin resulted in blood cell aggregates being trapped in small venules and capillaries in the ocular conjunctival vasculature [147]. In dogs, a serotonin antagonist prevented an increase in pulmonary alveolar dead space, an indication of pulmonary vascular obstruction, after hemorrhagic shock [261].

Several studies have found that SSRIs, including FLV, sharply reduce serotonin levels in blood, with reductions in plasma serotonin levels to 20–40% of baseline values over two to eight weeks being typical after the start of SSRI treatment [262,263,264,265,266,267,268]. All of these studies used high-performance liquid chromatography (HPLC) or enzyme-linked immunosorbent assay (ELISA) methodology for detection of serotonin plasma levels to avoid potential skewing of results from platelet uptake of serotonin [256,267]. For FLV in particular, mean plasma serotonin levels were reduced to 69% of the baseline value one hour after first dose of the drug [263]. A study of blood from humans and mice found that serotonin induced platelet aggregation [269] and platelet aggregation by arachidonic acid was decreased by 68% (*p* = 0.00001) in patients taking an SSRI vs. controls [270].

### 7.2. Hydroxychloroquine (HCQ)

The application of HCQ, an aminoquinoline, for treatment of COVID-19, as developed by an infectious disease team at Aix-Marseilles University in France [271,272,273], has been the subject of significant controversy, a review of which is not attempted here. However, it is of note that HCQ has been found to have pronounced activity in reducing blood cell aggregation and associated microvascular occlusion. In 44 human subjects with vascular conditions including coronary artery and cerebrovascular disease, all having initial manifestations of microvascular occlusion, ocular conjunctival microvasculature was observed over a nine-month period following the start of HCQ treatment [274]. Marked reductions in the size of blood cell aggregates and the extent of microvascular occlusion were observed for most patients. Accompanying symptomatic improvements were observed in many of these subjects beginning three days after the start of HCQ treatment for some and persisting over the nine-month follow-up period.

In another human study, HCQ was administered over a three-month period to 22 patients with rheumatoid arthritis who had signs of occlusion in the microcirculation of the ocular fundus. Twenty of the 22 patients had complete normalization of the observed vasculature occlusion [275]. In mice previously injected with an RBC clumping agent, HCQ sharply reduced thrombus size and the time that thrombi persisted as compared with untreated controls [276].

### 7.3. Ivermectin (IVM)

To identify potential therapeutics for COVID-19, four in silico studies collectively screened over 1000 molecules for binding to SARS-CoV-2 SP and other SARS-CoV-2 viral targets [105,277,278,279]. In each of these studies, the strongest or close-to-strongest binding affinity to SP was obtained for IVM, a macrocyclic lactone with multifaceted antiparasitic and antimicrobial activity, distributed in four billion doses for human diseases worldwide since 1987 [280,281,282]. Aminpour et al. (2022) found by molecular docking computations that IVM binds with high affinity (<−7.0 kcal/mol) to seven sialoside-binding sites or other glycan-binding sites on SARS-CoV-2 S1, six on the N-terminal domain (NTD) and one on the receptor-binding domain (RBD). These binding energy values of <−7.0 kcal/mol were obtained for the RBD in both the open (“up”) and closed (“down”) positions [99]. As a measure of significance of this binding energy value, binding energies of <−7.0 kcal/mol predicted efficacy for a large set of HIV inhibitors with 98% sensitivity and 95% specificity in another study [283]. Additional molecular modeling studies of IVM binding to SARS-CoV-2 SP [284,285,286,287,288], including one by Lehrer and Rheinstein (2020) [289], likewise found strong binding affinities for IVM.

Competitive binding by IVM to SP glycan-binding sites is thus a likely biochemical mechanism for the in vitro inhibition and reversal by IVM of aggregation of human RBCs by SARS-CoV-2 SP as noted above [91]. In early 2020, two Florida physicians, Jean-Jacques and Juliana Rajter, were intrigued by an indication of a clinical parallel to this in vitro effect—observations that several COVID-19 patients with severe respiratory impairment and SpO2 deficits experienced normalized breathing function within 1–2 days after treatment with IVM [290,291]. Months later, Rajter et al. (2020) reported results of a propensity-matched case control study of COVID-19 patients treated with a low dose of IVM plus standard of care (SOC) at four Florida hospitals, which yielded a 40% reduction in mortality vs. controls given only SOC [292]. After that study was concluded, through mid-2021, more than 20 randomized controlled trials (RCTs) of IVM treatment of COVID-19 were conducted, with six of seven meta-analyses reporting notable reductions in deaths and with a mean 0.31 relative risk of mortality vs. controls (a 69% reduction) [293].

By 2022, however, several other RCTs reported that IVM yielded no statistically significant benefits for COVID-19 treatment, as summarized in an August 2022 editorial which declared that it was “time to stop using ineffective COVID-19 drugs” [294]. Curiously, however, the editorial prominently cited in support a June 2022 meta-analysis of ten RCTs for IVM treatment of COVID-19 encompassing 3472 patients [295] which actually reported as the first finding in its results section a twofold reduction in deaths in its pooled IVM treatment vs. placebo groups (pooled log odds ratio of −0.67, 95% CI = −1.20 to −0.13, with low heterogeneity). The mortality reduction was less (log OR = −0.12) for the RCTs rated as having a low risk of bias, but included in that group, weighted to account for 63% of that pooled log OR, was a study of dubious credibility.

Coauthors of the aforementioned study, the TOGETHER trial [296], have repeatedly refused to disclose four of their key outcome numbers, namely per protocol deaths and hospitalizations, treatment vs. placebo [297], which are of key importance given critiques of the primary outcome used in all arms of the TOGETHER trial by the U.S. Food and Drug Administration [298] and National Institutes of Health [299]. Instead, a TOGETHER trial coauthor directed inquiring scientists to the ICODA data repository, listed as the data source in the study’s data sharing statement [297,300]. After two months of futile attempts by scientists to obtain the data from ICODA, however, on 7 June 2022, an ICODA manager reported that the TOGETHER trial data were never held by that data repository and that she had instructed its authors to stop citing it as their data source [297,301].

In another prominently cited RCT of IVM treatment of COVID-19 that reported negative conclusions [302], IVM was substituted for placebo doses for 38 patients, a mistake caught a month later, and blinding was broken by use of sugar water as the placebo for one-third of the study’s patients (liquid IVM has a bitter taste). Adverse events that are distinctive to the high IVM dose used (transient and non-critical) occurred at almost identical rates in the placebo and IVM arms, while over-the-counter sales of IVM surged in the study region during the study period [303].

The RCT evidence for IVM-based treatments of COVID-19 is thus mixed; however, in rare cases, efficacy of a drug has been conclusively established without RCT findings when it has achieved consistent major clinical benefits in the face of an established baseline of null effect. For example, the 96% cure rate for peptic ulcers by a triple therapy achieved in a 1990 clinical trial [304] provided conclusive evidence of the therapy’s efficacy, given a baseline of palliative but rarely curative results for that chronic condition [305,306]. The associated discovery of *H. pylori* as the underlying pathogen for peptic ulcers was honored with the Nobel prize for medicine in 2005 [307]. For penicillin, early in vitro and mouse studies provided convincing indications of marked antibacterial activity. Alexander Fleming found in 1929 that transparent regions formed around penicillin embedded in agar plates of several species of cultured bacteria, indicating inhibition of bacterial growth [308]. In a 1940 study, almost all penicillin-treated mice survived when exposed to bacterial strains that consistently caused fatal infections in untreated control mice [309]. In the absence of RCT evidence, penicillin production was then ramped up to industrial scale, saving the lives of thousands of soldiers during World War II [310].

**Figure 5:**
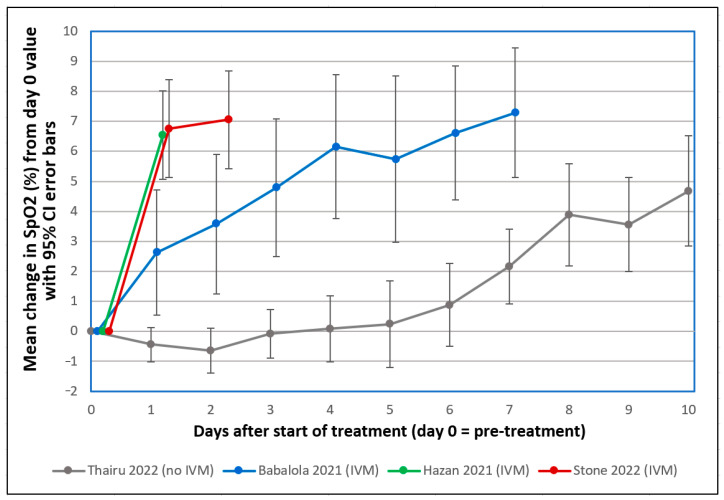
Mean changes in oxygen saturation (SpO2) for severe COVID-19 patients following treatments including or excluding IVM. Reproduced from Stone et al. (2022) [318] (CC-BY 4.0). Patients tracked over various time periods from each regimen were those with SpO2 values all recorded on room air, having pre-treatment (day 0) values ≤ 93%. The y-axis value at day n is the mean of changes in SpO2 values from day 0 to day n, with error bars designating 95% confidence intervals. ● Thairu et al. (2022) [321,322]: 26 patients, median age 45 years, treated with different combinations of lopinavir/ritonavir (Alluvia), remdesivir, azithromycin and enoxaparin as well as zinc sulfate and vitamin C. ● Stone et al. (2022) [318]: 34 patients, median age 56.5, treated with IVM, doxycycline and zinc. ● Hazan et al. (2021) [319]: 19 patients, median age 63, treated with IVM, doxycycline and zinc. ● Babalola et al. (2021) [320,321]: 19 patients, median age 33, treated with IVM, zinc and vitamin C, with some also given azithromycin and hydroxychloroquine.

For cases of moderate and severe COVID-19 in patients on room air, there is a consistent baseline of null effect in a 1–2 week timeframe: the magnitude of reductions in SpO2 levels correlate with the extent of pulmonary damage, and neither of these normalize in that timeframe [311,312,313,314,315,316,317]. With that backdrop of null effect, as shown in Figure 5, three studies of severe COVID-19 patients on room air treated with IVM-based regimens observed sharp increases in SpO2 after 1 day of treatment [318,319,320,321] while SpO2 decreased during the same 1-day period in a fourth group of such patients under standard care. The two studies that used the triple therapy of IVM, doxycycline and zinc [318,319], one of these coauthored by Thomas Borody [319], who developed the successful triple therapy for *H. pylori* [304], showed the most pronounced effect. For each of these three studies using IVM-based treatments, SpO2 changes one day after treatment differed from those values for a comparison study of COVID-19 patients on room air under standard care [321,322] with differences far outside the 95% confidence intervals for treatment vs. control values.

For the Stone et al. (2022) study, taking into account some missing values for the 34 treated patients at <48 h post-treatment, paired t-test calculations were performed for post-treatment minus pre-treatment SpO2 values for the study patients at +12 h, +24 h and +48 h after the start of IVM administration. These paired t-test values were highly significant, with *p* < 10^−6^ in each case. One patient in the study had an increase in SpO2 from 79% recorded at the first IVM dose to 95% three hours later, and four other patients had increases of 12 or more in SpO2% within 12 h after the first IVM dose. These sharp, rapid improvements parallel the disaggregation of RBC clumps observed in vitro over the course of 30 min by Boschi et al. (2022) and can be explained by rapid clearance of RBC aggregates in the vasculature and corresponding increases in efficiency of oxygenation in pulmonary and extrapulmonary tissues.

In 2020, Peru provided a unique setting to track clinical efficacy of IVM-based treatment for COVID-19 with close consideration of confounding factors, using excess deaths data from its national health system, which aligned with WHO monthly summary data [323]. Treatment with IVM and adjunct agents was deployed at intensive, moderate or limited levels under semi-autonomous policies in its 25 states, enabling comparisons with reductions in excess deaths at 30 days after peak values, state by state. A Kendall tau calculation yields a two-tailed *p*-value of 0.002 for reductions in excess deaths correlated with level of IVM use in Peru’s 25 states. On a national scale, during four months of IVM use in 2020, before a new president of Peru elected on November 17 restricted its use, there was a 14-fold reduction in nationwide excess deaths, and then a 13-fold increase in the two months following the restriction of IVM use [323]. This set of real-world national health data, accompanied by extensive additional data by which potential confounding influences can be tracked, provides another significant indication of efficacy of IVM treatment of COVID-19.

## 8. A Comparison of Degree of Clinical Susceptibility to COVID-19 and RBC Aggregability in Various Animal Species

Susceptibility to COVID-19 and severity of this disease have been tracked for dozens of mammalian species, as reported in a summary figure by Meekins et al. (2021) [324]. RBC aggregability values and related values of blood viscosity at low shear velocity have been tracked for many mammalian species as well, as reported by Baskurt and Meiselman in 2013 [325]. A correlation calculation between these two values, by species, provides a test of whether RBC aggregability is likely associated with COVID-19 morbidity.

The COVID status of mammalian species was reported by Meekins et al. using designators for viral shedding, clinical signs, mortality and transmission. We derived a composite COVID status index from the first three of these indicators (transmission was not used) with values of 0 for none of these three, 1 for viral shedding only, 2 for clinical signs and 3 for clinical signs and mortality. For RBC aggregability, an aggregation index shown in Baskurt and Meiselman for 22 mammalian species was used. They also reported values of blood viscosity under low-shear conditions for 27 mammalian species that were closely correlated with the corresponding RBC aggregation index for species having values shown in both figures. For a species tracked in Baskurt and Meiselman that reported blood viscosity but not RBC aggregability, the latter value was interpolated from the blood viscosity value. Correspondence between RBC aggregability and blood viscosity was established using the values of each for cattle and horses; these species had the minimum and maximum values of all species tracked by Baskurt and Meiselman, respectively, for both of these indices.

Table 1 shows the COVID status index and the RBC aggregability index, as described above, for the 13 species as tracked by both Meekins et al. and Baskurt and Meiselman, with the following adjustments: For the White-Tailed Deer as listed in Meekins et al., the mean of the RBC aggregation indices as interpolated from viscosity values for H. Deer, P.D. Deer and S. Deer (21.6, 23.5 and 9.1, respectively) reported by Baskurt and Meiselman was used. The contrast between high RBC aggregability in athletic species including horse, leopard and rhinoceros vs. low RBC aggregability in sedentary species including domestic cattle, sheep and goats has been noted by several observers [326,327,328], including Baskurt and Meiselman [325], who furnished these values for all four species. The susceptibility of domestic sheep and goats, neither tracked by Meekins et al., is consistently reported to be the same (minimal [329,330]) as that for domestic cattle, and the COVID status index for these two species, 0—the same as the Meekins et al. value for domestic cattle—was, therefore, added.

Using the methodology described above to determine indices for COVID status index and RBC aggregation for 13 matching mammalian species, the Kendall tau two-tailed rank coefficient was calculated [331]; this statistical test was selected because COVID status was meaningful as a ranking rather than a numerical measure. This calculation demonstrated a moderately significant correlation (*p* = 0.033, τb = 0.52), which could be interpreted to indicate that RBC aggregation is a key determinant but not the exclusive causal factor for COVID-19 morbidity in mammals.

## 9. Discussion

Consistent with coronavirus and RBC biochemistry established over past decades, the findings presented here demonstrate the central role of attachments from SARS-CoV-2 SP to sialylated glycans on RBCs and other blood cells in the severe morbidities of COVID-19. The glycans that decorate the SP of a coronavirus serve, metaphorically, as the virus’s arms and legs, its appendages of initial attachment to a host cell. The RBC, with its million strands of GPA per cell, along with platelets, offers an “immune adherence” defense of pathogens which can bind to glycans [72,82,83,84,85,86,87,88]. The associated hemagglutination is observed for many strains of coronaviruses [30,32,35,36,37,38,39,41,42], including SARS-CoV-2 [91].

Although these hemagglutinating properties of coronaviruses have been closely studied and the only known role of the GPA molecule on the RBC, the most abundant cell in the human body [332,333], is for pathogen binding and clearance [71,72,83,84], these glycan attachments have been largely overlooked in SARS-CoV-2 research. It is well established that RBCs, platelets and endothelial cells, which play key roles in COVID-19, are densely coated with sialylated glycans [64,65,70] but have no ACE2 (or, for endothelial cells, minimal ACE2 [69]) and that the various coronavirus strains use several different host-cell receptors for replication [30], yet ACE2 has been the exclusive host-cell target of interest in much of the research on SARS-CoV-2.

One explanation for this limited focus on the RBC and its pathogen-snagging GPA strands, one million per RBC, may be the lack of consensus on a solved structure of the extracellular domain of GPA [334,335]. Obstacles to this determination have been the extensive glycosylation of GPA, hindering the formation of a stable crystal for X-ray crystallography, and its intrinsically disordered structure [336,337] which allows a set of variable, extended and unfolded conformations [338,339,340].

An overemphasis on the role of viral replication and associated viral load in the pathology of SARS-CoV-2 has led to questionable conclusions. As noted, for the five human betacoronaviruses, the two benign and three deadly strains are distinguished not by viral load, which is about the same for the two common cold strains and SARS-CoV-2 [55], but by the expression of the enzyme HE, which releases glycan attachments to viral SP, only in the common cold strains, not in SARS, SARS-CoV-2 and MERS [50,51,52,53,54]. For an agent for which competitive binding to SARS-CoV-2 SP glycan-binding sites has been indicated in silico [99], IVM, one RCT tested it at a single low dose given on day 1 together with three other prophylactic regimens, each given daily for 42 days for prevention of COVID-19 infection [341]. The study concluded that IVM was ineffective because it yielded no significant reduction in viral load vs. controls, yet IVM at that single dose reduced the incidence of symptomatic COVID-19 and acute respiratory distress syndrome (ARDS) each by half, with associated *p* values of 0.0034 and 0.012, respectively [341].

Among the multifaceted demonstrations that SARS-CoV-2 SP-induced RBC aggregation and associated microvascular occlusion and hypoxia are central to severe morbidities of COVID-19, particularly informative are the countervailing effects of agents that inhibit glycan bindings of SP to RBCs. A mixture of heparan sulfate and heparin, both of which have strong binding affinity to SARS-CoV-2 SP [103,104,105], markedly reduced SARS-CoV-2 SP-induced thrombosis in zebrafish [103]. As noted, the strongest or close-to-strongest binding affinity to SARS-CoV-2 SP in molecular modeling screenings of more than 1000 total molecules was found for IVM [105,277,278,279]. Just as LMWD rapidly reversed HMWD-induced RBC aggregation in vitro [163] and in vivo [148,155,156,157,158], IVM both blocked and reversed SARS-CoV-2 SP-induced hemagglutination in vitro [91]. This effect was paralleled in three clinical studies as shown in Figure 5, in which depressed SpO2 values in severe COVID-19 patients on room air were sharply increased within 1–2 days [318,319,320] after the first IVM dose, in many cases within hours [318], in contrast to a null effect under SOC treatment in the fourth study shown.

Neither fibrin-hardened blood clots nor the blockage of all blood flow in a small-diameter capillary by RBC clumps would be readily reversible by clump disaggregation, even if effectively achieved. Observations of the reversal of HMWD-induced blood cell clumping by LMWD, however, provide insights into how disaggregation of RBC clumps by agents that competitively bind to SARS-CoV-SP could rapidly normalize blood flow and oxygen levels in severe COVID-19 patients. In mammals, a distributed network of arterioles can hold a significant total mass of RBC clumps before obstruction of blood flow becomes critical, while a pulmonary catch-trap architecture can also sequester large blood cell aggregates [167,178]. The dynamic, reversible character of RBC clumps in vivo up to a point at which the extent of aggregation becomes critical is demonstrated in the LMWD disaggregation studies noted above. A direct in vitro parallel, as noted, is the reversal of hemagglutination induced by SARS-CoV-2 SP over the course of 30 min by IVM in vitro [91]. A similar effect is strikingly demonstrated in the hemagglutination assay for viruses that express an enzyme (HE or similar) that cleaves host cell glycans. An interlaced sheet of RBCs initially forms and then subsequently collapses as that enzyme breaks the glycan attachments between viral SP and RBCs [31,32].

Although the central role of sialylated glycan bindings between SARS-CoV-2 SP and RBCs in the severe morbidities of COVID-19 has been the focus of this paper, such SP bindings to the heavily sialylated platelets and endothelial cells (which have no ACE2 and minimal ACE2, respectively) also contribute significantly to these morbidities, as noted above. Of particular interest is extensive damage to endothelial cells in severe COVID-19 patients, with an associated presence of SARS-CoV-2 virus and SP and elevated levels of VWF. As noted, an SA-cleaving enzyme was found to remove more than 50% of the glycocalyx of human kidney endothelial cells [70].

This examination of attachments from SARS-CoV-2 SP to sialylated glycans of RBCs and other blood cells and endothelial cells was spurred in part by an examination of possible molecular mechanisms of IVM activity in COVID-19 treatment and prevention. This may seem curious, given a general perception that IVM is ineffective against COVID-19 [294], yet major irregularities in some of the best-known such studies with negative conclusions, as noted in Section 7.3, indicate that the RCT evidence is more accurately characterized as mixed. It was also noted that in rare cases, such as for the triple therapy for peptic ulcers and for penicillin, striking demonstrations of drug efficacy against a consistent baseline of null effect under standard care established drug efficacy without accompanying RCT evidence. The findings of four studies depicted in Figure 5 appear to present a similar decisive demonstration of efficacy of IVM in treatment of pre-Omicron COVID infections.

The reports of distinguished scholars of scientific integrity, including current and past editors of leading scientific journals [342,343,344,345,346,347], on the vulnerability of science to commodification [343,347] and “flagrant conflicts of interest” [342] are also useful to bear in mind as this evidence is sorted out. As one case in point, although the triple-therapy cure for *H. pylori* was rapidly deployed in Australia, preventing an estimated 18,665 deaths there between 1990 and 2015 [348], it was not widely used in the rest of the world until the late 1990s, after the patents for the two best-selling palliative drugs for that condition had expired [349].

It is important to note, in evaluating drug treatment options for evolving COVID-19 variants, that Omicron viral strains, which became predominant in early 2022 [350], replicate less efficiently in the lung alveolar epithelium as compared with prior variants, in contrast to Omicron’s faster replication in the bronchi [351,352]. The disruption of the alveolar–capillary barrier is a prime route by which SARS-CoV-2 enters the blood stream [353], so limited replication of Omicron in alveolar tissue would limit viral loads in blood with associated reductions in RBC clumping and disease severity as caused by Omicron vs. prior variants. Thus, although Boschi et al. (2022) reported a tenfold greater hemagglutinating activity of Omicron as compared with prior variants [91], this would not appear to increase the severity of clinical infections, yet could possibly affect the incidence of adverse effects of COVID-19 booster vaccines for the Omicron variant, which have not been tested on human subjects [354]. Also, due to limited penetration by Omicron into the bloodstream, drugs that offer clinical benefits through reductions in RBC aggregation for pre-Omicron SARS-CoV-2 variants may not have significant efficacy against the less severe Omicron infections.

This has implications, for example, for evaluation of RCTs for FLV treatment of COVID-19, given that two recent such studies had substantial numbers of Omicron patients among their subjects [355,356]. On the other hand, IVM may maintain clinical efficacy against Omicron variants of SARS-CoV-2 though molecular mechanisms besides competitive inhibition of glycan bindings. For example, high-energy binding by IVM to the alpha-7 nicotinic acetylcholine receptor (α7nAChr), the main receptor activating the cholinergic anti-inflammatory pathway controlled by the vagus nerve [99,357], was predicted in silico [99] and was confirmed experimentally in both human and animal cells [358]. Activation of the α7nAChr by IVM has been demonstrated to trigger a marked increase in Ca++ current evoked by acetylcholine (e.g., a 20-fold shift in the affinity of acetylcholine [358]) and, accordingly, may dramatically decrease excessive macrophage inflammation and tumor necrosis factor (TNF), which play a major role during the inflammatory phase of COVID-19 infection (i.e., the cytokine storm) [99,357,359]. IVM binding to α7nAChr could also competitively inhibit viral penetration of macrophages and neuronal, endothelial and type II alveolar epithelial cells through this receptor [99,357].

For long COVID-19 patients, the demonstrated persistent presence of SP and subunits in plasma [111,112,113] and monocytes [114], respectively, and microvascular occlusion as seen in their sublingual vasculature [203] indicate an active therapeutic opportunity for drugs that limit SARS-CoV-2 SP binding to RBCs. Both optical coherence tomography angiography (OCT-A) and videomicroscopic imaging of the sublingual microvasculature offer tools to track microvascular occlusion that typically occurs in long COVID patients and to track any improvements that may be provided by drugs, either those highlighted here or others, in clinical treatment as well as in research settings.

## 10. Conclusions

The central role of sialylated glycan attachments between SARS-CoV-2 SP and RBCs and other blood cells in the severe morbidities of COVID-19 is founded on well-established biochemistry of coronaviruses and RBCs and established here through multiple channels of substantiation. Many preclinical and clinical studies show that SARS-CoV-2 SP attaches to and aggregates RBCs. Experimentally induced RBC clumping in vivo causes the same morbidities and the same redistribution of blood flow from smaller to larger blood vessels as for severe COVID-19. The key risk factors of increased age, diabetes and obesity for COVID-19 morbidity are each associated with significantly increased RBC aggregation. SARS-CoV-2 SP in the absence of whole virus as generated experimentally by IV injection of mRNA COVID vaccines in vivo, which caused SP to be generated in the absence of whole virus, induced microvascular occlusion.

Three generic agents which attracted prominent interest as COVID-19 therapeutics all yielded significant reductions in RBC aggregation. For mammalian species, the degree of clinical susceptibility to COVID-19 correlates with the aggregation propensity of RBCs with *p* = 0.033. These in vitro, in vivo and clinical findings, together, provide a convincing demonstration that RBC aggregation induced by SARS-CoV-2 SP through sialylated glycan attachments and resulting microvascular occlusion is key to the morbidities of severe COVID-19. These insights can support therapeutic and preventative strategies for evolving variants of this disease and for long COVID, while imaging of the retinal or sublingual microvasculature of active or long COVID patients can provide important support to these efforts.

## Figures and Tables

**Table 1:** Indices of COVID-19 status and RBC aggregability for mammalian species.

Species	COVID Index	RBC Aggregation Index
Domestic cat (Cat)	1 (V)	38.18
Malayan Tiger (Tiger)	2 (VC)	35.10
Lion (Lion)	2 (VC)	37.58 *
Snow Leopard (Leopard)	2 (C)	50.12 *
Domestic Dog (Dog)	0	28.15
White-Tailed Deer (H. Deer, P.D. Deer, S. Deer) **	1 (V)	18.06 *
Domestic Cattle (Cattle)	0	1.34
Domestic Pig (Pig)	0	30.27
House Mouse (Mouse)	0	0.18
Cottontail Rabbit (Rabbit)	0	5.20
Common Marmoset (Marmoset)	2 (C)	3.40 *
Sheep, domestic livestock (Sheep) ***	0	0.18
Goat, domestic livestock (Goat) ***	0	0.18
KENDALL TAU	τb = 0.52	*p* = 0.033

COVID index from Meekins et al. (2021) [324], with RBC aggregation index for the matching species (listed in parentheses) from Baskurt and Meiselman 2013 [325]. For COVID index, V = viral shedding, C = clinical signs; no matching species here was reported as having mortal cases. * Value was interpolated from low-shear blood viscosity. ** RBC aggregation index is the mean of those for the three deer species listed. *** COVID index values for these species were added as commonly reported in other sources [329,330].

## Data Availability

Not applicable.

## Abbreviations

The following abbreviations are used in this manuscript:

α7nAChr	alpha-7 nicotinic acetylcholine receptor
ARDS	acute respiratory distress syndrome
BMI	body mass index
CEC	circulating endothelial cell
COVID-19	coronavirus disease 2019
ECG	electrocardiogram
ELISA	enzyme-linked immunosorbent assay
FDG	fluorodeoxyglucose F18
FLV	fluvoxamine
Gal	galactose
GPA	glycophorin A
HCQ	hydroxychloroquine
HE	hemagglutinin esterase
HMWD	high-molecular-weight dextran
HPLC	high-performance liquid chromatography
IM	intramuscular
IV	intravenous
IVM	ivermectin
LMWD	low-molecular-weight dextran
long COVID	post-acute sequelae of COVID-19 or PASC
MW	molecular weight
Neu5Ac	α5-N-acetylneuraminic acid
NTD	N-terminal domain
OCT-A	optical coherence tomography angiography
PBS	phosphate-buffered saline
RBC	red blood cell
RBD	receptor-binding domain
RCT	randomized controlled trial
SA	sialic acid
SARS-CoV-2	severe acute respiratory syndrome coronavirus 2
SOC	standard of care
SP	spike protein
SpO2	peripheral oxygen saturation
SSRI	selective serotonin reuptake inhibitor
VD	vascular density
VWF	von Willebrand factor
